# The Role of Efferocytosis in Autoimmune Diseases

**DOI:** 10.3389/fimmu.2018.01645

**Published:** 2018-07-20

**Authors:** Fereshte Abdolmaleki, Najmeh Farahani, Seyed Mohammad Gheibi Hayat, Matteo Pirro, Vanessa Bianconi, George E. Barreto, Amirhossein Sahebkar

**Affiliations:** ^1^Cellular and Molecular Research Center, School of Paramedical Sciences, Qazvin University of Medical Sciences, Qazvin, Iran; ^2^Department of Genetics and Molecular Biology, Isfahan University of Medical Sciences, Isfahan, Iran; ^3^Department of Genetics, School of Medicine, Shahid Sadoughi University of Medical Sciences, Yazd, Iran; ^4^Unit of Internal Medicine, Angiology and Arteriosclerosis Diseases, Department of Medicine, University of Perugia, Perugia, Italy; ^5^Departamento de Nutrición y Bioquímica, Facultad de Ciencias, Pontificia Universidad Javeriana, Bogotá, Colombia; ^6^Instituto de Ciencias Biomédicas, Universidad Autónoma de Chile, Santiago, Chile; ^7^Biotechnology Research Center, Pharmaceutical Technology Institute, Mashhad University of Medical Sciences, Mashhad, Iran; ^8^Neurogenic Inflammation Research Center, Mashhad University of Medical Sciences, Mashhad, Iran; ^9^School of Pharmacy, Mashhad University of Medical Sciences, Mashhad, Iran

**Keywords:** apoptosis, efferocytosis, autoimmune disease, phagocytosis, systemic lupus erythematous

## Abstract

Apoptosis happens continuously for millions of cells along with the active removal of apoptotic debris in order to maintain tissue homeostasis. In this respect, efferocytosis, i.e., the process of dead cell clearance, is orchestrated through cell exposure of a set of “find me,” “eat me,” and “tolerate me” signals facilitating the engulfment of dying cells through phagocytosis by macrophages and dendritic cells. The clearance of dead cells *via* phagocytes is of utmost importance to maintain the immune system tolerance to self-antigens. Accordingly, this biological activity prevents the release of autoantigens by dead cells, thus potentially suppressing the undesirable autoreactivity of immune cells and the appearance of inflammatory autoimmune disorders as systemic lupus erythematous and rheumatoid arthritis. In the present study, the apoptosis pathways and their immune regulation were reviewed. Moreover, efferocytosis process and its impairment in association with some autoimmune diseases were discussed.

## Introduction

During the lifespan of the human body, a huge number of cells die after fulfilling their functions in each tissue (1). In this regard, apoptosis is a highly organized process of cellular suicide and programmed cell death, which is critical to control body homeostasis and to regulate tissue development (2). The removal of cell corpses through different signaling is performed by phagocytic and dendritic cells of the innate system in physiological conditions. Immediately after apoptosis, phagocytes are recruited by appropriate “find me” signals and, after “eat me” signals are released, they may finally engulf the apoptotic cells (3). The appropriate operation of cell corpse clearance, termed as efferocytosis, is imperative for organ development, tissue adjustment, and accomplishment of a proper immune response (4). Failure in the process of dead cell clearance may lead to various disorders, including autoimmune diseases such as systemic lupus erythematous (SLE), rheumatoid arthritis (RA), and type 1 diabetes (T1D) (5, 6). Thus, a growing interest has mounted in recent years on the mechanisms, modulation, and correlation of dead cell clearance in specific illnesses (4). In this review, the molecular pathways occurring during apoptosis, the role of efferocytosis as a modulator of immune response and the mechanisms leading to the omission of cell corpse removal by professional and non-professional phagocytic cells are outlined.

## Apoptosis Mechanism

Apoptosis, as a homeostatic process, happens during development and aging in order to promote embryogenesis and maintain cell population in organs and tissues (7). Numerous studies employing different techniques such as the terminal deoxynucleotidyl transferase (TdT) dUTP Nick-End Labeling (TUNEL) assay and real-time qPCR have revealed that such a natural mechanism is characterized by the activation of different pathways (e.g., caspases pathway) and several morphological changes including cell shrinkage, blebbing, chromatin condensation, and DNA fragmentation (8, 9). Finally, the apoptotic process should be terminated by the phagocytosis of the apoptotic corpses, which are enveloped with their entire plasma membrane. Immediate phagocytosis of these apoptotic cells by macrophages prevents cell secondary necrosis and the release of cellular debris, thus avoiding inflammatory reactions, as well as anti-inflammatory cytokines production (10).

As anticipated, one of the most important feature of apoptotic pathways is the presence and activation of caspase proteins. Such proteins are expressed in the form of an inactive proenzyme in most cells, but their activation induces the initiation of a protease cascade leading to apoptotic signaling pathway as well as rapid cell death (7). There are different kinds of caspases endowed with various potentials including initiator caspases (2, 8, 9, and 10 caspases), effector or executioner caspases (3, 6, and 7 caspases), as well as inflammatory ones (1, 4, and 5 caspases). Besides, all of them have a common feature, that is, a proteolytic activity to separate proteins at aspartic acid residues (11). Apoptotic cells have also some biochemical characteristics to be recognized by phagocytes in order to have minimum engagement with their surrounding tissues. These features include: (1) the translocation of phosphatidylserinee (PS) to the external layer of the plasma membrane, (2) and the expression of calreticulin (CRT) and annexin 1 proteins on the surface of apoptotic cells (12). CRT, as a second general recognition ligand, can be recognized by an LDL receptor-related protein on the engulfing cells, together with the interaction between the apoptotic and engulfing cells mediated by the general recognition ligand PS, which colocalizes with CRT on the surface of apoptotic cell and acts as a detection signal for phagocytes. Hence, the interaction of annexin V as a recombinant PS-binding protein with PS residues is able to facilitate the recognition of apoptosis and the combined action of PS and CRT is important for optimal apoptotic-cell recognition and uptake of apoptotic cells (13).

Two main pathways of apoptosis include extrinsic or death receptor pathway and intrinsic or mitochondrial pathway, which are induced *via* extracellular signaling and mitochondrial proteins, respectively (14). To initiate the apoptosis process *via* an extrinsic pathway, two central receptor-mediated interactions are involved: fatty acid synthase ligand and receptor (FasL/Fas R) and tumor necrosis factor-alpha and receptor (TNF-α/TNF R) (15). Binding of FasL to Fas R can lead to the constitution of the death-inducing signaling complex containing Fas-associated death domain protein (FADD), caspase 8, and caspase 10. This process is followed by the activation of executioner caspases 3, 6, and 7, as well as the induction of cell death (16). In addition, signaling through TNF ligand to TNF R can result in the binding of the adaptor protein TNF receptor-associated death domain (TRADD) utilizing FADD and receptor-interacting protein and inducing apoptosis in a caspase-independent manner (16, 17).

The main function of the intrinsic apoptosis pathway is related to mitochondria. Various factors including DNA damage, hypoxia, radiations, heat, and viral infections may cause mitochondrial swelling and membrane permeabilization, followed by leaking out of apoptotic effectors (18, 19). Such a mechanism starts *via* releasing the second mitochondria-derived activator of caspases (SMAC) into the cytosol and enhancing the permeability of the mitochondria membranes. Moreover, SMAC is able to deactivate the proteins hindering apoptosis (IAPs), thus allowing apoptosis to continue (20). In another way, due to the formation of a channel in the outer membrane of mitochondria, that is the mitochondrial apoptosis-induced channel (21), cytochrome C is extracted, thus promoting caspase 9 activation and related morphological changes in association with apoptosis (e.g., changes in the nucleus, DNA fragmentation, PS appearance on the cell surface). Accordingly, binding of cytochrome C to apoptotic protease activating factor-1 (Apaf-1) and ATP can lead to its connection to pro-caspase9 in order to produce a set of proteins known as apoptosomes. The activity of apoptosome can also change the procaspase to its active form (caspase9) followed by the activation of effector caspase-3 (18). Mitochondrial functions during apoptosis can be also controlled and regulated *via* members of B-cell lymphoma protein 2 (BCL-2) protein family (22). The BCL-2 family members include both proapoptotic proteins [e.g., BCL2-associated X protein (Bax), BCL2 antagonist killer 1 (Bak), BH3-interacting domain death agonist (Bid), BCL2 antagonist of cell death (Bad), and BCL2-interacting protein BIM (Bim)] and antiapoptotic proteins [BCL-2, BCL2 related protein, long isoform (BCL-xl), BCL2 related protein, short isoform (BCL-xs), and BCL2 associated athanogene (BAG)]. These proteins are of particular importance in order to determine cell fate, i.e., apoptosis or prevention of the mechanism. In this sense, the release of cytochrome C from the mitochondria to the cytosol can be regulated by changing the mitochondrial membrane permeability *via* BCL2- family proteins (7).

There is a connection between the extrinsic and intrinsic apoptosis pathways. First, the activation of the extrinsic pathway by Fas domain leads to the activation of Bid to Truncated Bid (tBid) *via* caspase 8-mediated cleavage. Then, tBid binds and inhibits Bcl-2 and also stimulates oligomerization of Bax or Bak, leading to the release of cytochrome C and the activation of the internal pathway (23, 24).

### Apoptotic Versus Non-Apoptotic Cell Death

In addition to programmed cell death (i.e., apoptosis), accumulating evidence has led to a better comprehension of additional types of cell death, including necrosis, necroptosis, pyroptosis, and ferroptosis. Different stimuli including ischemia, pathogens, irradiation, heat, or cytokines may promote necrosis. Since necrosis is a passive mode of cell death, it has been suggested initially that there was no specific mechanism in association with necrotic cell death; however, it is now clear that necrotic cell death has multiple subtypes, greatly organized by particular molecules. For instance, mixed lineage kinase domain-like, receptor interacting serine/threonine kinase 1, receptor interacting serine/threonine kinase 3 (RIPK3) have been established to contribute to a subtype of necrosis termed necroptosis (25), whereas another subtype of necrotic cell death, i.e., pyroptosis, is in association with caspase-1-mediated cell death (26). An additional subtype of non-apoptotic cell death requiring iron ions is named ferroptosis (27). It was revealed that these new subtypes of cell death, albeit sharing some common mechanisms, may be differently regulated. However, there is not enough evidence on the physiological activity of these new subtypes of cell death and on the differential response of macrophages and dendritic cells to these types of cell death. In addition, it is still unclear whether the specific form of cell death can determine the procedure of dead cell clearance.

## Mechanism of Efferocytosis

Several billion cells are dying in the human body to ensure cellular homeostasis, wound healing, and immune responses. In order to allow all these processes, the dying cells should be efficiently removed (28). The process of dead cell clearance, termed as efferocytosis, is normally done in an orchestrated mechanism in the human body through lifespan (29). Due to the released signals from dead cells and phagocytes, both professional (i.e., macrophages and dendritic cells) and non-professional cells (i.e., epithelial cells and fibroblasts) participate actively to apoptotic corpses identification and engulfment in order to degrade them (30, 31). The engulfment process of apoptotic cells by phagocytes, before apoptotic cells release their immunogenic intra-cellular contents, is considered as an immunological event. Accordingly, impairment of this process can lead to numerous autoimmune disorders such as SLE, RA, and other diseases (32). Altogether, the efferocytosis process can be classified into four steps: (1) the leaking of the “find me” signal by dead cells to recruit phagocytes, (2) phagocyte identification and its contribution to the “eat me” signals on the cellular corpse, (3) the engulfment of dead cells, and (4) the degradation of the engulfed cells (3).

### “Find-Me” Signals

Accumulating evidence demonstrated that the dying cells are able to reveal their presence to phagocytes (33). Specifically, based on the acquired data from *Caenorhabditis elegans*, it was realized that recruitment of phagocytes to the area of cell death can happen before cell apoptosis is completed (34, 35). Expression of various “find me” signals by apoptotic cells can attract phagocytic cells through a chemotactic gradient (36). Four main “find me” signals have been described, including nucleotides, CX3CL1, lysophosphatidylcholine (LPC), and sphingosine-1-phosphate (S1P).

The caspase-dependent release of nucleotides as ATP and UTP through pannexin-1 (panx1) channels is believed as a crucial “find me” signal (37), which can result in warning phagocytes to cooperate with purinergic receptors (e.g., P2Y2) and removal of dead cells (33).

During maturation of B cells, lots of them will be apoptosed and release the membrane-associated molecule CX3CL1 (fractalkine), whose recognition by CX3CR1 can modulate the migration of macrophages toward the dying B cells. Although experiments conducted on mice with CX3CR1 deficiency shed light on the migration of macrophages toward apoptotic B cells, the mechanisms of apoptotic B cell clearance by phagocytic cells needs to be clarified (38).

Lysophosphatidylcholine, which is another “find me” signal, is produced and released through caspase-3-dependent activation of phospholipase A2. There is evidence that ATP-binding cassette transporter A1 may be necessary for LPC release by apoptotic cells (39). This lipid signal is mediated by the G-protein-coupled receptor G2A on macrophages (40). The other lipid “find me” signal, released by dead cells, is S_1_P, which is generated from sphingosine by sphingosine kinase and sensed *via* multiple G-protein-coupled receptors (i.e., S_1_P-R1-5) to regulate phagocyte chemotaxis (41).

In comparison with necrosis or necroptosis, the discharge of nucleotides from apoptotic cells is small and the released nucleotides can be easily degraded by extracellular nucleotidases (36). In addition to the release of a low amount of extracted nucleotides (<2% of intra-cellular ATP) (33), the “find me” signals of lipid origin during apoptosis all act in a short-range to engage phagocytes (32).

There are additional issues that make the efferocytosis process even more intricate. For instance, apoptotic cells may release also lactoferrin glycoprotein that acts as a “keep me” signal and refuses neutrophils and eosinophils from the area of cell death (42, 43). Thus, the balance between “find me” and “keep me” signals might be crucial for the final destination of the apoptotic debris. Another finding in this domain is the double role of the “find me” signal as danger-associated molecular pattern to activate innate immune system (44) or stimulating factors to prime phagocytes (45).

**Figure 1:**
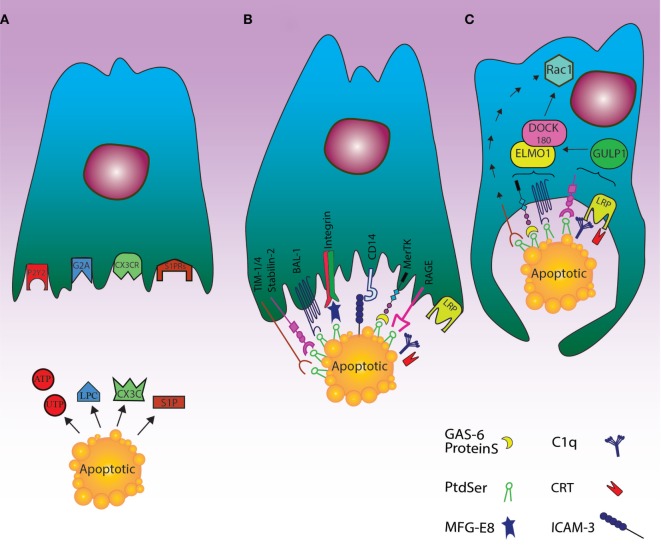
Cell death clearance processing by phagocytes through finding apoptotic cells, recognition their signals and engulfment of the cell corpses. **(A)** The “find me” signal: the dying cells release signals such as UTP, ATP, sphingosine-1-phosphate (S1P), and lysophosphatidylcholine or fractalkine through apoptosis. These “find me” signals can also conduct phagocytes to the location of cell death. Phagocytes can thus sense the “find me” signal to detect apoptotic cells using cognate receptors including sphingosine-1-phosphate receptor (S1PRs), purinergic receptors (P2Y2), G-protein-coupled receptor (G2A), and CXCR3. **(B)** The “eat me” signal: the dying cells expose “eat me” signals on their surface, so phagocytes can recognize and engulf apoptotic cells by recruiting a complex of receptors and bridging molecules. The main common “eat me” signals include the expression of phosphatidylserine (PS) on the outer layer of plasma membrane, brain-specific angiogenesis inhibitor 1, T cell immunoglobulin mucin receptor (TIM1, TIM3, TIM4), RAGE, and stabilin along with PS-specific bridging molecules, Gas6, Milk Fat Globule EGF Factor 8 (MFG-E8), and protein S. Other “eat me” signals include calreticulin (CRT) and ICAM3, which can modulate the identification and engulfment of apoptotic cells by LRP receptors (*via* C1q) and CD14, respectively. **(C)** The engulfment process: after recruitment of engulfment receptors through the activity of Rac pathway, the polymerization of actin and rearranging of cytoskeletal are initiated. Although the mechanism of TIM4 in this process is unknown, some engulfment receptors recruit the DOCK180/ELMO1 set (αvβ3, TAM, stabilin-2, and LRP). Thus, disorders during this step can lead to autoimmunity and inflammatory.

In conclusion, since dying/dead cells together with healthy cells and immune cells coexist in the body, the phagocytes should be able to distinguish dying/dead cells from living ones, while dying/dead cells should display specific signals in order to be differentiated from living cells and to be engulfed by phagocytes (46, 47), thus preventing undesirable inflammation and ensuring tissue homeostasis (Figure 1A). Although some functions of the reported “find me” signals have been clearly described (e.g., producing cells, releasing pathways, target cells), there are still several open questions regarding the possible interaction (either positive or negative) between both “find me” and “keep me” signals and target cells. First, it is unclear whether “find me” and “keep me” signals may be cell-specific, so that different dying cells may preferentially recruit different phagocytes. Second, it is not established as to whether different “find me” and “keep me” signals at various concentrations and distances from phagocytes may induce different effects. Third, the function of metabolites deriving from the extracellular breakdown of “find me” signals within the microenvironment surrounding dying cells needs to be clarified. Additional research addressing these issue might provide a better understanding of the impact *in vivo* of these signals both in physiological and pathological conditions.

### “Eat-Me” Signals

The most effective “eat me” signal in well-organized efferocytosis is the extra-cellularly exposed lipid PS. The asymmetrical distribution of lipids in plasma membrane is well recognized; thus, PS is normally present in the inner leaflet in living cells, whereas it is expressed externally during the apoptotic process in a caspase-dependent manner (48). The mechanism for this translocation also includes the TMEM16F as a calcium-mediated cation channel, which can moderate lipid scrambling (49). On the other hand, the cleavage of the scramblase XKr8 by caspase-3 can facilitate the expression of PS on the extra-cellular side of plasma membrane (50). In particular, it has been found that apoptotic stimuli may promote the formation of a plasma membrane complex between XKr8, basigin, and neuroplastin, which is required for the scrambling activity of XKr8 (51). Also, the activity of flippase ATP11C in transferring the aminophospholipids from outer leaflet to inner one during apoptosis is suppressed by caspase-3 cleavage, which can result in extra-cellularly PS exposure (52). PS, as the most critical “eat me” signal, has multiple receptors to be recognized by phagocytes including bona fide membrane receptors such as stablin-2 (53), RAGE (54), TIM4 (in addition to family members of TIM1 and TIM3) (55, 56), and brain-specific angiogenesis inhibitor 1 (57). Moreover, additional factors participate in PS recognition and dead cell engulfment including a number of bridging molecules like proteins, Gas6 (58), and MFG-E8 (59), as well as the associations between MFG-E8 and integrin αvβ3 or αvβ5 and those between Tyro3-Axl-Mer (TAM) family of receptors and proteins and Gas6 (60), CD14 and ICAM3 (61), and scavenger receptors like SR-A and oxidized LDL-like moieties (62). Also, the interaction between external CRT with complement C1q (63), and glycosylated surface protein with lectin (64) could be considered as additional contributor signals for apoptotic cell clearance. In particular, CRT may be expressed by apoptotic cells after a sequence of key events in the dying cells, including endoplasmic reticulum stress, eIF2alpha phosphorylation, caspase8 activation, Bap31 cleavage, and Bax activation (65). The interaction between apoptotic cell surface CRT and phagocyte CD91 is then followed by apoptotic cell phagocytosis.

While exposure of PS is found in low levels on living cells, the presence of signals such as CD31, CD47, and CD61 on their surface is considered as a “do not eat me” signal for not being engulfed by phagocytes (66, 67). In this regard, the balance between “eat me” and “do not eat me” signals can more effectively and actively regulate the clearance of dying cells by phagocytes (5). There is also a cooperation between dying cells and phagocytes, so that cellular corpses can advertise their desire to be engulfed by phagocytes by expressing “find me” and “eat me” signals; on the other hand, the phagocytes can prompt their engulfment through employing receptors that distinguish these signals (3) (Figure 1B).

Overall, the role of the complex interplay between “eat me” and “do not eat me” signals and the pathological consequences of their derangement are far from being well understood. Further studies aimed at mapping with molecular probes the crucial players of the entire phagocytosis pathway induced by both “eat me” and “do not eat me” signals might help to elucidate this issue.

### Phagocytosis of Cellular Corpse

The expression pattern, modes of identification, and downstream signaling of PS receptors are different. Beside professional phagocytes, PS receptors are expressed in different tissues including lungs (RAGE), kidneys (TIM-1), spleen (BAI-1), bone marrow (BAI-1), brain (BAI-1), and sinusoidal endothelium (stabilin-2) (36). There is an association between receptors and tissue specificity, explaining that different tissues need specialized PS receptors for efficient efferocytosis (3). For instance, expression of a defective BAI-1 in glial and neuronal cells can lead to apoptotic corpses accumulation and neurodegenerative disorders (68).

It has been shown that PS can be recognized by various domains of the above mentioned molecules (55). For example, stabilin-2 uses its EGF-like domains for PS identification (53), TIM receptors use their Ig-variable (IgV) domain (55) and MFG-E8 its C1 and C2 discoidin-like domains (69). Beside these, extracellular signaling (between PS and bridging molecules), intracellular signaling cascades are necessary to facilitate the engulfment of dead cells (70). Molecules such as Rho family of small GTPases involving Rock, Rac, RhoA, Rab 5, and CDC42 are also engaged to mediate the absorption of dead cells (71). These molecules are moderated between an inactive GDP-bound state and an activated GTP-bound one by definite guanine-nucleotide-exchange factors as the bipartite GEF constituted by DOCK 180 and ELMO1 (72). The process of clearing the dead cells by phagocytes *via* active membrane disruption is also like macropinocytosis process (73), which is different from complement-receptor-mediated phagocytosis, so that the engulfment of corpse by negative regulation of RhoA is inhibited *via* its overexpression and it is dependent on Rho-associated coiled-coil-containing protein kinase (ROCK) (74). Using phosphorylation of myosin light chain, ROCK kinase activity can also induce actomyosin accumulation and cell contraction (73). Thus, the decrease of RhoA activation can result in the reduction of signaling by ROCK, lessening of stress fiber constitution, and preparing cell shape changes for efficient engulfment (74).

Unlike RhoA, the function of Rac1 in an evolutionarily preserved pathway is critical for developing phagocytic potential (71) since Rac1 activation can induce polymerization of actin and arrangement of cytoskeletal through Scar/WAVE composite (75, 76). Although the mechanism of CDC42 in the engulfment of dead cells by phagocytes is not precisely cleared (77), enclosing within the phagocyte and the downstream events of this metabolic pathway are started altogether (Figure 1C).

### Digestion and Immune Response

The apoptotic cellular corpse, as an ingested cargo, includes several compounds (e.g., lipids, proteins, and others) that can force the phagocytes to remove them in an immunologic procedure (14). The fusion of the phagosomes with the lysosomes can lead to the activation of lysosome enzymes such as acid proteases and nucleases to destruct the apoptotic cell constituents. However, this process may lead to local inflammation. Thus, for instance, DNAse II is necessary for DNA degradation, a process that can be followed by aggregation of DNA fragments within phagocytes, which in turn may promote inflammation and polyarthritis (78, 79).

In order to maintain tissue homeostasis and to prevent inflammation, phagocytes with engulfed dead cells can produce anti-inflammatory cytokines, including TGFβ and interleukin-10 (80). The engulfment of apoptotic cells may also suppress pro-inflammatory cytokines (e.g., TNF-alpha, IL-1, IL-12) (81). In addition, *via* the activation of peroxisome proliferator-activated receptor γ/δ, as an important regulator of cellular lipid homeostasis and apoptotic cell clearance, cholesterol can suppress inflammatory responses (82).

To manage the process of degradation of the cells, at least two definite pathways for engulfment of external or internal unwanted particles have been described, including phagocytosis and autophagy (83). More recently, LC3-associated phagocytosis (LAP) has been proposed as a novel non-canonical process of autophagy involving the clearance of extracellular apoptotic debris and pathogens. LAP, as a process combining the preserved pathways of phagocytosis and autophagy machinery, may be induced when extra-cellular molecules including pathogens, dead cells, or immune complexes are recognized by extra-cellular receptors such as toll-like receptor 1/2 (TLR1/2), TLR2/6, TLR4, FCR, and TIM4 and result in the application of some members of the autophagy machinery to the cargo-containing vesicle (84).

**Figure 2:**
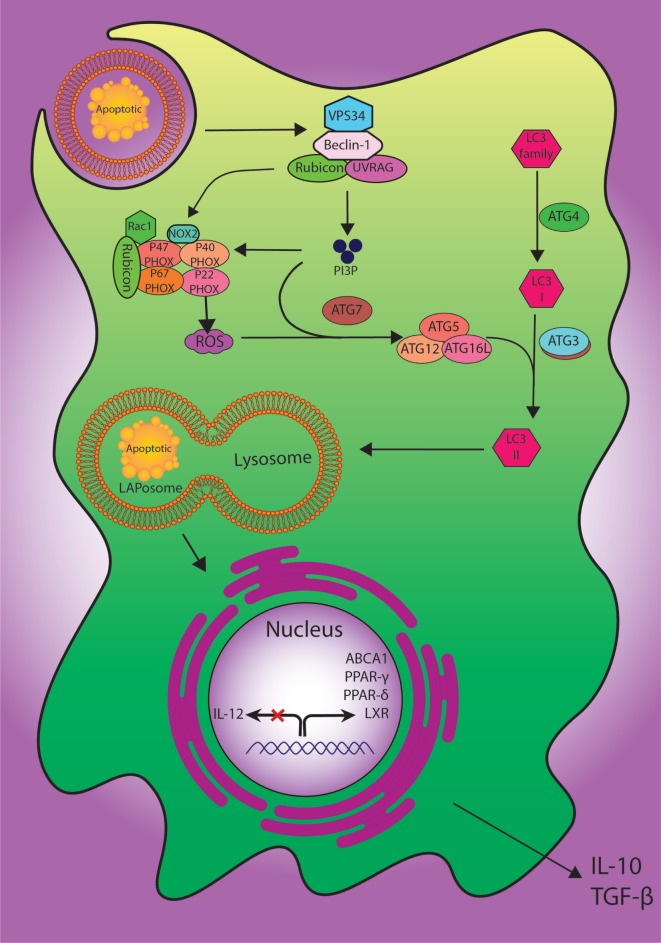
The digestion process of engulfed dead cells. During the engulfment of apoptotic cells, LC3-associated phagocytosis (LAP) pathway accompanied by its components and ULK1/2 complex as a canonical autophagy pathway have specific roles. The class III PI3K collection involving VPS34, Beclin-1, UVRAG, and Rubicon are also important to the localization of PI3P at the LAPosome and canonical autophagy. PI3P has two functions: the employment of the downstream autophagic/LAP machinery including ATG5, ATG7, ATG12, and ATG16L, and fixation of the NOX2 set with recruitment of Rubicon. Also, merging the lysosome and LAPosome maturation is in need of LC3-II. The cholesterol sensors such as liver X receptor, ABCA1, PPARδ, PPARγ, and PGC-1β are similarly required for anti-inflammatory impact of efferocytosis, which can result in producing anti-inflammatory modulator such as IL-10 and TGF-β.

In spite of having common molecular machineries, there are multiple features to discriminate LAP from autophagy based on the structure of LC3-decorated phagosome (or LAPosome). It was elucidated that autophagosomes involve a bilayer membrane to surround autophagic cargo, whereas the LAP consisted of a monolayer membrane structure (85, 86). The formation of autophagosomes also takes long hours, whereas the phosphatidylinositol 3-phosphate (PI3P) activity in L3-decorated phagosome (LAPosome) is performed in a few minutes after phagocytosis (86, 87). Autophagy is also dependent on pre-initiation complex including FIP200, ULK1/2, and ATG13 while LAP functions do not (84). Both autophagy and LAP require the class III PI3kinase complex and its core components of VPS34, VPS15, and Beclin-1; besides, LAP can recruit the UVRAG containing class III PI3kinase complex (88). The defensive role of LAP against autoimmune responses has also been established; accordingly, its failure can lead to a sustained pro-inflammatory status (86). However, the regulation of immune responses *via* LAP pathway needs further studies to be more clearly delineated (Figure 2).

## Pathologies Associated with Improper Efferocytosis

Apoptosis, as a well-organized mechanism, can play a critical role to adjust immune system and homeostasis. Thus, even a small failure in this multi-step procedure can lead to many disorders. If an apoptotic cell is not removed in a specific period of time, it is possible to be ruptured and finally dispense its harmful contents. Since these released particles are considered as autoantigens, they result in the promotion of immune responses (89, 90). In this respect, accumulating evidence demonstrates that impairment in the process of efferocytosis is straightly linked to the appearance of numerous autoimmune and inflammatory diseases such as SLE, RA, type I diabetes, multiple sclerosis (MS), and others, which are delineated as follows (91).

### Systemic Lupus Erythematous

One of the autoimmune diseases that is strongly associated with impaired efferocytosis is SLE. This disease can cause a wide range of clinical symptoms through the involvement of skin, joints, kidney, lungs, nervous system, heart, and blood vessels (92). Since unengulfed apoptotic cells exist in the germinal centers of the lymph nodes of some patients with SLE and the extracted macrophages from these patients can reveal low ingestion ability of apoptotic cells, the failure of dead cells clearance can be attributed to be one of the reasons of SLE (93). Moreover, SLE patients have circulating autoantibodies against nuclear antigens [e.g., antinuclear antibodies (ANA) and anti-DNA antibodies] (94). Thus, the binding of autoantibodies with autoantigens leads to the constitution of immune complexes, which can participate to the etiopathogenesis of SLE-associated nephropathy (95).

Previous experiments conducted on mice with deficiency in one of the various receptors of efferocytosis including MFG-E8, BAI-1, TIM-4, or MerTK have demonstrated that dead cells were concentrated in their lymph nodes. In addition, they suffered from an SLE-like disease with splenomegaly and glomerulonephritis manifestations (45, 96, 97). These mice also generated a high level of anti-double-stranded DNA and antinuclear antibodies. As well, abnormal splicing of MFGE-8 and failure of C1q complements in MFG-E8-deficient mice resulted in impaired engulfment of dead cells, secondary necrosis following the release of cellular compartments and production of autoantibodies (45). In this regard, mice affected with defective MFG-E8 are considered as good models for investigating the molecular process in which endogenous cellular constituents can stimulate the immune system extra-cellularly (45).

As explained above, at the early stage of programmed cell death, the apoptotic cells are distinguished and engulfed by macrophages mostly due to a PS-dependent way. Nevertheless, the mechanism of recognizing and engulfing necrotic cells *via* macrophages is not completely clarified. It has been hypothesized that the complement system may have a role in this process (98). At the early stage of apoptosis, C1 connects to dead cells through IgM-dependent manner and *via* the expression of LPC signals on them for IgM-binding (99). It is also verified that humans with deficient C1q gene are prone to develop SLE. In mice with C1q deficiency like MRL/Mp strain, unengulfed dead cells stimulate the development of SLE-like glomerulonephritis (100). It is interesting that some polymorphism in ATG5 (101) and likely ATG7 genes (102), engaged in both autophagy and LAP, have been identified through genomic studies as propensity markers for SLE (84, 85, 87).

Among the different organs potentially affected by SLE, heart involvement may be of particular severity. In particular, QT interval prolongation, which may be promoted by both cardiomyocyte apoptosis (103) and chronic inflammation (104), is a common finding in SLE (105) and may increase the risk of cardiovascular complications (106). Since enhanced efferocytosis of apoptotic cardiomyocytes promotes inflammation resolution and cardiac repair (107), this might translate into improved electric impulse propagation as well. However, a better understanding of the involvement of efferocytosis in arrhythmias and cardiac involvement in SLE is still needed.

### Anemia and Polyarthritis

When apoptotic cells are engulfed by phagocytes, they can be degraded within lysosomes into their constituents, including nucleotides, fatty acids, amino acids, and monosaccharides. DNA is also degraded within lysosomes *via* DNase II in acidic situations (108). DNase II is expressed in different tissues, especially in macrophages; therefore, insufficient DNase II in macrophages can result in the accumulation of nucleotides followed by production of cytokines by macrophages. IFNβ, the main kind of cytokines produced by macrophages, is cytotoxic to lymphocytes and erythroblasts (109). Specifically, *via* TUNEL assay on mice, it was demonstrated that erythroblasts could be killed by IFNβ function; thus, severe anemia may occur following IFNβ exposure. Furthermore, severe anemia is also the cause of death in mice with DNase II deficiency in embryogenesis (78). Moreover, mice with both deficiency at DNase II and IFN-type I receptor or mice with deleted genes of DNAse II through knockout procedure after birth could promote polyarthritis (79). RA is a systemic and chronic autoimmune disorder with inflammatory joint involvement, which can be manifested by developed circulating autoantibodies against citrullinated peptides or complement protein C3 and rheumatoid factor (110, 111). In the swollen joints of patients with RA, i.e., the site of joints with aggressive pannus formation and cartilage erosion, the genes of inflammatory cytokines such as IL-1β, IL-6, and TNFα are activated (112). Hence, RA patients can be treated *via* antagonism of these cytokines (79).

Experiments on DNase II null mice showed undigested DNA expressing TNF-α mRNA in macrophages, and the presence of low levels of TNF-α in serum before detecting any abnormalities in the joints. TNF-α production *via* macrophages can be responsible for the promotion of polyarthritis (113). Synovial cells also respond to TNF-α in order to generate IL-1β and IL-6, trigger the expression of TNF-α gene (114), and consequently produce cytokines in the joint, which can result in polyarthritis development (115).

Anemia and polyarthritis may be related to DNase II deficiencies as a more complex lysosomal storage disease, caused by malfunction of lysosomal enzymes including glycosidases, proteases, and lipases. As a consequence, DNA, RNA, proteins, and polysaccharides of bacterial or viral origin can stimulate the innate immunity and generate different cytokines (116). Obtained from DNase II null mice, accumulated DNA in the lysosome of mammalian macrophages can activate the innate immune responses. It is likely that other undegraded components in lysosome stimulate IFNβ and TNFα genes. Secretion of cytokines by macrophages that are deficient in the production of lysosomal acid lipase (117) and by fibroblasts of patients with Niemann–Pick Disease Type C (118) can consequently support this fact. Treatment of some polyarthritis patients with bone marrow transplantation confirms the presence of a deficiency in bone-marrow-derived cells in these patients. Besides, determining whether there is a defect in their lysosomal enzyme could also be useful to develop a treatment procedure (119). Although there is not enough genetic evidence to link human RA and efferocytosis, studies have revealed the possibility to increase the amount of bridging molecules for TAM receptor or stimulating the liver X receptor/PPARγ, which could have therapeutic advantages in mice with inflammatory arthritis (120).

### Type 1 Diabetes

Pancreatic insulin-producing B cell destruction is responsible for development of T1D, a T cell-mediated autoimmune disorder leading to insulin deficiency and hyperglycemia. It is believed that inefficient clearance of apoptotic pancreatic cells may promote the release of signals and autoantigens into the media *via* the creation of necrosis and inflammation (121). The lack of T cell tolerance to self-antigens is also a critical factor in T1D patients. Recently, some studies have demonstrated that defective clearance of dead cells is in association with immunogenic responses (not tolerogenic), maturation of DC, and chronic inflammation. In this respect, a study on non-obese diabetic (NOD) mice, which instinctively develop TID mellitus, showed that there were not only defects in the process of apoptotic cell clearance by phagocytes *in vitro*, but also the efferocytosis mechanism through apoptotic stimulation had its own deficiencies *in vivo*. Thus, impaired apoptotic cell clearance by NOD mice contributed to the generation of ANA (122).

A common feature among patients with both Type1 and Type2 Diabetes mellitus is the imperfect wound healing. It has been observed that aggregation of dead cells at the site of the wound can lead to inflammation and slow wound healing as a result of incomplete efferocytosis (123, 124). Although the relationship between diabetes and efferocytosis has been investigated, the absolute mechanism of the effect has not been still recognized.

### Multiple Sclerosis

Multiple Sclerosis is known as a chronic and degenerative disorder of the central nervous system (CNS), distinguished by focal lesions with inflammation, oligodendroglial death, demyelination, and axonal damage (125). These cellular changes are accompanied by neurological deficiencies such as sensory disruption, visual deficits, loss of motor regulation, and production of elevated level of IL-1β cytokine from monocytes and macrophages. MS is usually started with an autoimmune inflammatory response to myelin constitutions and develops to a chronic stage *via* degeneration of myelin, axons, and oligodendrocytes (126, 127). The most common reason for MS pathology is related to excitotoxicity, produced by primary and/or secondary changes in glutamate signaling (128). *Via* ATP as the main neurotransmitter in the CNS, ionotropic (P2X), and metabotropic (P2Y2) receptors are activated (129). Interestingly, both P2X and P2Y receptors have been involved in MS (130), with variable influence according to different P2X and P2Y subtypes. Furthermore, both P2X and P2Y have been found to recognize different “eat me” and “find me” signals during efferocytosis (33, 131). Thus, P2X and P2Y could be regarded as possible target in MS. However, it must be recognized that supportive data on how defective clearance of apoptotic neural cells contribute to MS pathogenesis are not still clarified. In particular, despite there is evidence that panx1 is the molecular substrate for P2X7 and P2X7 is involved in neuronal death and MS (132), the impact of P2X7 neuronal death on efferocytosis has not been defined. Hence, additional research is needed for a better comprehension on whether impaired efferocytosis may influence MS pathogenesis.

### Autoimmune Lymphoproliferative Syndrome (ALPS)

The ALPS (133, 134) disease is an autoimmune disease characterized by impaired lymphocyte homeostasis and increased susceptibility to malignancies (135). The disease is manifested by hypergammaglobulinemia, increased level of FAS ligand (136), and IL-10 (137) in plasma, along with accumulation of double-negative T cells (CD4^−^CD8^−^ T cells) (138). First, the disease was identified in an experimental mouse with FAS and FASL mutations (139), which are imperative for apoptosis mechanism. Moreover, it has been understood through further investigations on both mice and humans that the given factors as parts of the TNF receptor family are necessary for an apoptosis procedure in order to prevent the assembly of self-reactive T and B lymphocytes (140). In this regard, it is realized that defective apoptosis pathway can lead to the activation of immune system and manifest itself as a disease affecting other organs of the body due to mutation in FASL and deficit signaling pathway (134, 141). Hence, because FAS/FASL pathway may serve as a “find-me” signal in efferocytosis (142), it is arguable that mutations involving genes of this pathway, as observed in ALPS, might have also an impact on related diseases.

### Ulcerative Colitis

One of the forms of inflammatory bowel disease (IBD) is ulcerative colitis. The given disease is considered as a chronic and relapsing disorder of the large intestine manifested by contiguous inflammation of the colonic lamina propria (143). A critical factor in the pathogenesis of the IBD is host identification of bacteria, which are sensed by the immune system *via* specific receptors resulting in inflammation (144). Lipopolysaccharide (LPS), as the main component of cell wall in bacteria, is also recognized by TLR-4, and it can result in NF-κB-related stimulation of inflammatory responses (145). In this regard, mice with deficiency in TLR-4 have an impaired response to LPS. Since TLRs are key activators of innate immunity, insufficient activation of innate immune system *via* intestinal luminal antigen or deficient regulation of its signaling results in an imbalance between effectors and regulatory cells, leading to over-responsiveness to these bacteria and intestinal inflammation (146, 147). Besides TLRs, particularly TLR4, other molecules like LPS-binding protein and the bacterial permeability increasing protein (BPI) with a high affinity to LPS, have been reported in human serum (148, 149). These complexes, which are recognized by CD14, a glycosylphosphatidylinositol-anchored molecule on the monocyte surface, are activated in the form of potent antimicrobial proteins (150). Since CD14 as a bridging gap is in connection with ICAM3, facilitating the recognition and engulfment of apoptotic cells results in efficient efferocytosis.

It has been demonstrated that two mutations in extracellular domain of TLR-4 along with a polymorphism in the gene of CD14 contributed to ulcerative colitis (151). Moreover, it has been reported that individuals with both mutations of TLR-4 in the airway epithelia do not respond to the induction of LPS; thus, the reduced expression of TLR was seen on their apical surface (152), which resulted in increased risk of bacterial infections (153). It was also realized that deficient receptors on the cells could not activate the signaling cascades of immunity to clear the bacterial infection; thus, they resulted in immune responses and the IBD (154–156).

### Crohn’s Disease (CD)

A kind of chronic inflammatory disease of the gastrointestinal tract is CD, in which, altered immune response against intraluminal microbiota may occur in a susceptible host (157). There are several genes in association with CD, but one of the most important is the nucleotide-binding oligomerization domain containing 2 or (NOD2) gene, known as CARD15 (158). The Nod-protein family is comprised of intra-cellular and host-specific cytosolic pattern-recognition receptors with a significant role in innate immunity to detect pathogen-associated molecular patterns of different microorganisms in order to produce transcriptional responses against bacteria (159). In this regard, two genes can contribute to autophagy including autophagy-related 16-like 1 (ATG16L1) (160) and the immunity-related GTPase family M (IRGM) (161). Recent studies have also shed light on a relationship between Nod2 and autophagy pathway in which Nod2 recruits the ATG16L1 to the plasma membrane through the bacterial invasion; thus, patients with mutations in Nod2 have a deficiency in bacterial trafficking, autophagy induction, and antigen presentation, which result in steady inflammation (162). Beside these genes, the ULK1 is another autophagy gene contributing to CD. It has been determined that the silencing of ULK1 can inhibit autophagy that finally results in accumulation of dead cells, acute immune responses, as well as inflammation in the gastrointestinal tract (159).

## Conclusion

Apoptosis is a natural mechanism to maintain homeostasis and tissue development, which has been extensively studied. Due to the importance of programmed cell death, a deeper understanding of the mechanisms of this process, classification of its subtypes and pathological significance, as well as further comprehension of the relationship between diseases and cell death require additional clarification. However “postapoptotic biological events” have drawn the interest of many researchers. Indeed, the field of efferocytosis is rather young and numerous autoimmune disorders have been investigated to be correlated with impaired efferocytosis. However, there are many challenges in this field to be overcome such as the identification of various steps of efferocytosis mechanisms and their relation to diseases and understanding of the kinds of engulfing phagocytes and their relation to tissue specificity. Thus, the interest in examining efferocytosis and autoinflammatory disorders continues to grow. Precise understanding of efferocytosis and its biological and physiological roles in the autoimmune system can light new insights on how to prevent diseases in early stages and help with providing therapeutic methods to treat autoimmune disorders.

## Author Contributions

Discussed the review content: FA, SH and AS; wrote and critically revised the manuscript: FA, NF, SH, MP, VB, GB and AS; all authors agreed and approved the submission.

## Conflict of Interest Statement

The authors declare that the research was conducted in the absence of any commercial or financial relationships that could be construed as a potential conflict of interest.

## Abbreviations

BA1, brain-specific angiogenesis inhibitor 1; C1q, complement 1q; CRT, calreticulin; LPC, lysophosphatidylcholine; LXR, liver X receptor; MFG-E8, milk fat globule-EGF factor 8; PPARδ/γ, peroxisome proliferator-activated receptor γ/δ; RA, rheumatoid arthritis; SLE, systemic lupus erythematous; S1P, sphingosine-1-phosphate; ATG, autophagy-related gene; LAP, LC3-associated phagocytosis; TLR1/2, toll-like receptor 1/2; TIM, T cell immunoglobulin mucin receptor; DAMP, danger-associated molecular pattern.
